# Artificial intelligence-based personalised rituximab treatment protocol in membranous nephropathy (iRITUX): protocol for a multicentre randomised control trial

**DOI:** 10.1136/bmjopen-2024-093920

**Published:** 2025-04-02

**Authors:** Maxime Teisseyre, Alexandre Destere, Marion Cremoni, Kévin Zorzi, Vesna Brglez, Sylvain Benito, Laurent Bailly, Céline Fernandez, Barbara Seitz-Polski

**Affiliations:** 1 Institut de Recherche sur le Cancer et Vieillissement UMR7284 CNRS INSERM U1081, Université Côte d’Azur, Nice, Provence-Alpes-Côte d’Azur, France; 2 Centre de Référence Maladies Rares Syndrome Néphrotique Idiopathique et Glomérulonéphrite Extra-Membraneuse, Université Côte d’Azur, Centre Hospitalier Universitaire de Nice, Nice, Provence-Alpes-Côte d’Azur, France; 3 Département de Néphrologie, Dialyse et Transplantation, Université Côte d’Azur, Centre Hospitalier Universitaire de Nice, Nice, Provence-Alpes-Côte d’Azur, France; 4 Laboratoire d’Immunologie, Unité de Thérapie Cellulaire et Génique, Université Côte d’Azur, Centre Hospitalier Universitaire de Nice, Nice, Provence-Alpes-Côte d’Azur, France; 5 Département de Pharmacologie Clinique et de Pharmacovigilance, Université Côte d’Azur, Centre Hospitalier Universitaire de Nice, Nice, Provence-Alpes-Côte d’Azur, France; 6 Inria, CNRS, Laboratoire J.A. Dieudonné, Maasai team, Université Côte d’Azur, Nice, Provence-Alpes-Côte d’Azur, France; 7 EXACTCURE, Nice, Provence-Alpes-Côte d’Azur, France; 8 Département de Santé Publique, Université Côte d’Azur, Centre Hospitalier Universitaire de Nice, Nice, Provence-Alpes-Côte d’Azur, France

**Keywords:** Artificial Intelligence, IMMUNOLOGY, Nephrology, Machine Learning, Glomerulonephritis

## Abstract

**Introduction:**

Membranous nephropathy is an autoimmune kidney disease and the most common cause of nephrotic syndrome in non-diabetic Caucasian adults. Rituximab is now recommended as first-line therapy for membranous nephropathy. However, Kidney Disease Improving Global Outcomes guidelines do not recommend any specific protocol. Rituximab bioavailability is reduced in patients with membranous nephropathy due to urinary drug loss. Underdosing of rituximab is associated with treatment failure. We have previously developed a machine learning algorithm to predict the risk of underdosing. We have retrospectively shown that patients with a high risk of underdosing required higher doses of rituximab to achieve remission. The aim of this prospective study is to evaluate the efficacy of algorithm-driven rituximab treatment in patients with membranous nephropathy compared to standard treatment.

**Methods:**

A multicentre, randomised, controlled, open-label, prospective superiority clinical trial will be conducted in 13 French hospitals. 130 consecutive patients with primary membranous nephropathy and active nephrotic syndrome will be randomised to either the standard protocol control group (two 1 g rituximab infusions on days 0 and 15) or the algorithm-driven rituximab treatment group. In the latter, the rituximab dose will depend on the algorithm-estimated risk of underdosing. Patients with an algorithm-estimated risk of underdosing ≤50% will receive 1 g of rituximab on days 0 and 15. Patients with an algorithm-estimated risk of underdosing between 51% and 75% will receive 1 g of rituximab on days 0, 15 and 30. Finally, patients with an estimated risk of underdosing >75% will receive 1 g of rituximab on days 0, 15, 30 and 45. The primary study outcome is the rate of clinical remission (complete or partial) at month 6 after treatment initiation. The secondary outcomes include clinical remission at month 12, immunological remission, proteinuria, albuminuria, serum creatinine, estimated glomerular filtration rate, phospholipase A2 receptor type 1 antibody titre, anti-rituximab antibody occurrence, lymphocyte count, serum rituximab level and related adverse events.

**Ethics and dissemination:**

The trial received ethics approval from the local ethics boards. The results of this study will confirm whether algorithm-driven rituximab treatment is more effective in inducing remission than the standard regimen and thus may contribute to improving management of patients with membranous nephropathy. The results of our study will be submitted to a peer-review journal.

**Trial registration number:**

NCT06341205 trial number. Registered on 2 April 2024.

**Strengths and limitations of this study**

- The artificial intelligence-based personalised rituximab treatment protocol in membranous nephropathy (iRITUX) trial is a multicentre randomised controlled trial conducted in 13 centres in France.
- The iRITUX trial is the first study to evaluate the value of artificial intelligence to guide treatment in membranous nephropathy.
- The iRITUX trial is not a double-blind trial due to the different treatment regimens, but the results will be analysed blinded to treatment allocation.

## Introduction

 Membranous nephropathy is an autoimmune kidney disease that represents the most common cause of nephrotic syndrome in non-diabetic Caucasian adults. The characterisation of membranous nephropathy as an autoantibody-driven disease and the identification of podocyte target antigens represent major advances in the diagnosis and follow-up of patients. Phospholipase A2 receptor type 1 (PLA2R1), the main target antigen, is involved in 70%–80% of cases of primary membranous nephropathy.^1^ Anti-PLA2R1 antibodies result in *in situ* deposits of immunoglobulins along the glomerular filtration barrier, which can be observed by immunofluorescence.^2 3^ These immune complexes lead to the activation of the complement system and induce the glomerular lesions responsible for nephrotic syndrome.^3^,^6^ These findings have rationalised the use of B cell-depleting drugs such as rituximab. Rituximab is a chimeric monoclonal antibody that targets the CD20 antigen on B cells. Over the last decade, rituximab has emerged as a first-line therapy for membranous nephropathy with proven safety and efficacy, and it is now listed in the 2021 Kidney Disease Improving Global Outcomes (KDIGO) guidelines.^7^ However, several factors may limit the efficacy of rituximab in the treatment of membranous nephropathy: (1) irreversible chronic glomerular damage; (2) the presence of antidrug antibodies and (3) reduced bioavailability.^8^,^13^ In fact, in autoimmune diseases other than membranous nephropathy, residual rituximab serum levels can be detected for 6–9 months after the first infusion. This is due to the recycling from endothelial cells via the neonatal fragment crystallisable receptor (FcRn).^14^ However, rituximab may be eliminated in the urine of nephrotic patients, as demonstrated in the comparison of two cohorts of nephrotic and myasthenic patients, paired by age, sex and weight, treated with the same rituximab regimen (1 g, 2-week interval). Residual rituximab level at month 3 was statistically lower in nephrotic patients compared with myasthenic patients.^11^ Rituximab was also detected in the urine of nephrotic patients from the 15th day after infusion.^11^ This urinary drug loss reduces rituximab exposure and may explain why the rituximab regimen of two 375 mg/m^2^ infusions did not demonstrate efficacy at month 6 compared with a non-immunosuppressive antiproteinuric regimen in the GEMRITUX study (NCT01508468).^15^ In contrast, a regimen of two 1 g infusions 2 weeks apart was associated with a significantly higher remission rate at month 6 than the GEMRITUX regimen.^16^ This high-dose regimen was associated with higher residual serum rituximab levels, more efficient B cell depletion and faster depletion of anti-PLA2R1 antibodies.^16^

Recently, we have shown that after two 1 g infusions of rituximab, patients with higher residual serum rituximab levels 3 months after rituximab infusion were more likely to achieve clinical remission at 6 and 12 months.^10^ Patients with a serum albumin level below 22.5 g/L at baseline had an 8.66-fold increased risk of having an undetectable rituximab level at month 3, demonstrating that the more severe the nephrotic syndrome, the more undertreated the patient will be. Therefore, it appears that optimisation of the rituximab regimen in membranous nephropathy should be considered. Early additional doses of rituximab may be beneficial in patients with a higher risk of rituximab underdosing to improve treatment bioavailability and increase the likelihood of remission. Evaluating the best dose schedule for rituximab in membranous nephropathy is one of the research recommendations of the 2021 KDIGO guidelines.^6^ In addition, the 2021 KDIGO guidelines do not specify which of the rituximab regimens is to be preferred.^6^ Tailoring the rituximab regimen to patient characteristics is an important issue.^17^,^22^ Repeated doses of rituximab, up to cumulative doses of 3000–6000 mg, according to autoantibody immunomonitoring, have been shown to be effective in inducing clinical remission while being safe.^17 23^ There is only one randomised controlled trial (NCT03804359) comparing personalised treatment with rituximab to the standard protocol.^22^ In this trial, treatment was personalised according to the PLA2R1 epitope spreading.

Nowadays, machine learning algorithms are increasingly used in medicine and especially in pharmacology to predict the exposure to a drug, the initiation dose or the interval between two infusions. We have developed a machine learning algorithm to predict the risk of rituximab underdosing.^24^ Based on age, sex, body surface area, anti-PLA2R1 antibody titre at day 0, serum albumin at day 0 and at day 15 and serum creatinine at day 0 and at day 15, this algorithm demonstrated its ability to predict rituximab underdosing at 3 months with an accuracy, sensitivity and specificity of, respectively, 79.4%, 78.7% and 81.0%. To validate the clinical relevance of our algorithm, we analysed the outcome of 45 patients with membranous nephropathy treated with rituximab. We retrospectively analysed the cumulative doses of rituximab received to achieve clinical remission and the time to achieve clinical remission according to the initial probability of rituximab underdosing proposed by our algorithm. Patients with a high initial probability of rituximab underdosing (>50%) had a longer time to remission (probability of rituximab underdosing ≤50%: 4.2±1.8 months vs 51%–75%: 8.4±4.6 months vs >75%: 10.7±5.9 months; p=0.002) and received higher cumulative doses of rituximab (probability of rituximab underdosing ≤50%: 2.0±0.0 g vs 51%–75%: 2.7±0.9 g vs >75%: 3.6±1.6 g; p=0.001).^24^

To prospectively evaluate our algorithm, we planned a multicentre, randomised study to evaluate the superiority and safety of an algorithm-guided rituximab regimen versus a standard regimen in patients with membranous nephropathy.

## Methods and design

### Design

**Figure 1:**
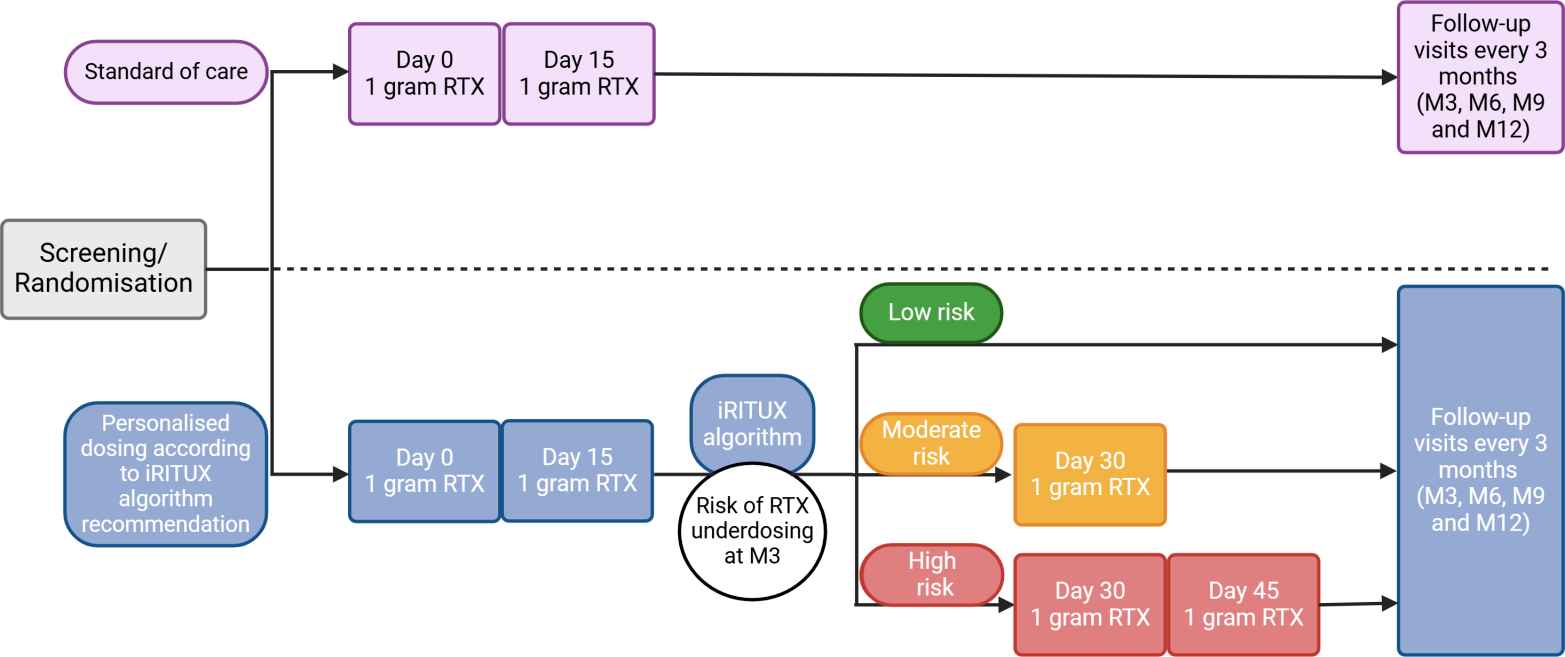
Detailed overview of the iRITUX study protocol. Created with Biorender.com. iRITUX, artificial intelligence-based personalised rituximab treatment protocol in membranous nephropathy; Mx, month-x; RTX, rituximab.

This is a parallel, two-arm, randomised, open-label, multicentre (13 sites in France), prospective study comparing the superiority and safety of an algorithm-guided rituximab regimen with the standard regimen in adult patients with membranous nephropathy. The study will last 7 years, with a 6-year enrolment period and 1 year of follow-up. An overview of the study is shown in figure 1.

### Coordinating centre

The coordinating centre consists of:

- A medical team (principal investigator (PI) and co-PI experts in membranous nephropathy) responsible for patient screening, recruitment and medical care. They are also responsible for validating the artificial intelligence-based personalised rituximab treatment protocol in membranous nephropathy (iRITUX) algorithm with a pharmacologist.
- A study coordinator is responsible for patient protocol visits, data entry and site coordination.
- A central laboratory team (one engineer and one technician) supervises sample collection and analysis for all sites.
- A sponsor team (project manager and research assistants) is responsible for all regulatory submissions, administrative coordination and monitoring.
- A central pharmacy specialising in clinical trials is responsible for shipping the investigational product to the sites.

### Inclusion criteria

Patients will be recruited if they fulfil the following criteria: age ≥18 years; membranous nephropathy diagnosed by either the presence of circulating anti-PLA2R1 or anti-thrombospondin type-1 domain-containing 7A (THSD7A) antibodies or kidney biopsy; active nephrotic syndrome defined by proteinuria >3.5 g/24 hours (or urine protein-creatinine ratio >3.5 g/g) and serum albumin <30 g/L at screening; estimated glomerular filtration rate (CKD-EPI formula) >30 mL/min/1.73 m^2^; indication for rituximab treatment according to the 2021 KDIGO and French guidelines; non-immunosuppressive antiproteinuric treatment at a stable dose for 2 weeks, including a renin-angiotensin-aldosterone system inhibitor, a diuretic and a low-salt diet at the maximum tolerated dose (ie, absence of orthostatic hypotension and no increase in serum creatinine >30%); social insurance; and signed informed consent (online supplemental file 1). Women of childbearing age must use an effective method of contraception up to 12 months after the last rituximab dose is received.

### Exclusion criteria

Patients with any of the following conditions will be excluded: secondary membranous nephropathy (related to cancer, infection, systemic lupus or drugs); pregnancy or breastfeeding; immunosuppressive treatment (including rituximab) in the 6 months prior to enrolment; the presence of anti-rituximab antibodies detected by the central laboratory; cancer under treatment; active serious infections; hypersensitivity to the active substance or excipients; severely immunocompromised patients who, in the opinion of the investigator, cannot receive more than two 1 g doses of rituximab; severe congestive heart failure or severe uncontrolled heart disease; persons deprived of liberty or incapacitated adults; patients who refuse to follow the algorithm recommendation approved by the referring nephrologist; and patients who, in the opinion of the investigator, cannot receive the dose regimen recommended by the algorithm (for tolerance or compliance reasons).

### Randomisation

After signing the informed consent and completing the screening visit to validate the inclusion criteria, participants will be randomised to one of the two treatment arms: (1) standard rituximab regimen and (2) algorithm-guided rituximab regimen. Centralised block randomisation will be balanced (1:1). Randomisation will be integrated into the electronic case report form (e-CRF) specifically designed for the study using the Research Electronic Data Capture (RedCap) software. Entering their personal access details to log in, the investigators will provide the necessary patient information (ie, the first letter of their first and last name and their month and year of birth) for random assignment to treatment using an online randomisation module (RedCap). The treatment group and inclusion number for the patient will be relayed to the investigator and the central laboratory team. The patient’s trial records will then be created automatically, allowing data to be entered.

### Investigational product

Celltrion Healthcare will provide rituximab (Truxima®) free of charge for this study. The investigational rituximab will be stored in a secure area in accordance with local regulations.

### Machine learning algorithm

A machine learning algorithm was developed to predict the risk of rituximab underdosing (concentration <2 µg/mL at month 3).^24^ The algorithm is based on a support vector machine (SVM) with a polynomial kernel and was selected after comparison with several supervised models, including logistic regression, random forests and artificial neural networks. The SVM was chosen for its ability to handle data with small sample sizes while maximising the separation between classes in a high-dimensional space.

The performance of the models was assessed by 10-fold cross-validation on the training set (75% of the data). The SVM achieved an accuracy of 79.4%, outperforming logistic regression (70.6%), random forests (73.5%) and artificial neural networks (68.3%). In terms of sensitivity and specificity, the SVM showed a balanced performance (84.6% and 72.7%, respectively, on the test set) compared with the results obtained by logistic regression (sensitivity 75% and specificity 65%) and random forests (sensitivity 78.1% and specificity 68.2%). The SVM also showed an F-score of 81.5%, highlighting its overall effectiveness in categorising patients.

The model’s input variables were selected using a forward-backward approach, systematically assessing the impact of adding or removing a variable on predictive performance. The variables selected included age, sex, body surface area (BSA), anti-PLA2R1 antibody titre at day 0 (optional variable), serum albumin and serum creatinine at days 0 and 15. Prior to model training, data were preprocessed with K-nearest neighbour imputation of missing values, followed by standardisation to ensure homogeneous scaling of continuous variables.

### Treatment arms

#### Experimental strategy

For patients randomised to the experimental arm, the iRITUX multidisciplinary staff will run the algorithm and make a recommendation to the referring nephrologist based on the risk of rituximab underdosing at month 3.

- Patients with a risk between 0% and 50% will receive 1 g×2 (day 0 and day 15).
- Patients with a risk between 51% and 75% will receive 1 g×3 (day 0, day 15 and day 30).
- Patients with a risk between 76% and 100% will receive 1 g×4 (day 0, day 15, day 30 and day 45).

If the opinion of the referring nephrologist contradicts the result generated by the iRITUX algorithm, the opinion of the patient’s treating nephrologist will prevail in all cases.

#### Control strategy

Patients randomised to the control arm will receive two courses of rituximab at a dose of 1 g on days 1 and 15.

To improve compliance, patients will be contacted by telephone before each treatment. A medical consultation is also scheduled for all rituximab injections.

### Relevant concomitant care

Medications not listed in the exclusion criteria may be administered at the investigator’s discretion. The investigator will record all concomitant medications taken by the participant in the appropriate section of the case report form.

### Sample size

Sample size calculation was performed according to the sequential design method in order to consider the implementation of three interim efficacy analyses. The sample size was calculated with SAS applying the SEQDESIGN procedure, considering the risk of increasing the alpha risk due to multiple analyses. The method used in our case was the O’Brien-Fleming type of boundary (design method=obf) to maximise our chance of stopping the study at the final analysis, if we cannot reach a positive conclusion during the interim analyses.

The criteria applied for the sample size calculation were a total alpha risk at 0.025, with an estimated result of 75% remission in the personalised arm (number of rituximab doses defined by the algorithm) versus 50% in the standard arm (rituximab 1 g×2) and a one-sided sample test for the difference between two binomial proportions. These assumptions about remission rates are based on observations from a retrospective cohort comparing remission rates in patients with rituximab underdosing with those in patients without rituximab underdosing.^10^

Finally, to maintain the total alpha risk at 0.025 and the total beta risk at 0.2 with four analyses, we need to enrol 130 patients in our study, which accounts for a 5% rate of lost to follow-up (anticipated as minor in this study as patients are intensively followed for this pathology).

### Evaluation criteria

The primary endpoint is the clinical remission 6 months after the first rituximab infusion. Clinical remission is defined as a composite criterion, combining according to KDIGO guidelines: (1) complete clinical remission, defined as urinary protein/creatinine ratio (UPCR) <0.3 g/g in a morning spot urine sample and serum albumin >30 g/L and (2) partial clinical remission, defined as UPCR <3.5 g/g with a decrease >50% from baseline (ie, first rituximab infusion) and improvement or normalisation of serum albumin.

Secondary endpoints include complete clinical remission at month 12; partial clinical remission at month 12; immunological remission (ie, negative antibody titre by ELISA or negative indirect immunofluorescence) at month 3, month 6, month 9 and month 12; changes in proteinuria and albuminuria from baseline to month 3, month 6, month 9 and month 12; changes in serum creatinine and CKD-EPI estimated glomerular filtration rate from baseline to month 3, month 6, month 9 and month 12; changes in anti-PLA2R1 antibody ELISA titre from baseline to month 3, month 6, month 9 and month 12 (patients with PLA2R1-associated membranous nephropathy only); occurrence of anti-rituximab antibodies at month 3, month 6, month 9 and month 12; residual serum rituximab level at month 3 after the first rituximab infusion; recording adverse events related to treatment during study follow-up; changes in lymphocyte counts: B cells (CD19, transitional, mature and memory) and T cells (CD3, CD4, CD8 and T-Reg); changes in non-immunosuppressive antiproteinuric treatment during study follow-up; assessment of clinician satisfaction with the algorithm used to calculate the risk of rituximab underdosing (in the experimental group only); predictive rate (%) of the algorithm for rituximab underdosing in the control group; pharmacokinetics in all patients with serum creatinine and serum albumin levels, weight, anti-PLA2R1 and rituximab level at day 0, day 15, day 30, day 45, month 3 and month 6; and plasma cytokine levels after non-specific stimulation of innate and adaptive immunity cells in pg/mL (interferon (IFN)-γ, IFN-α, interleukin (IL)-12p70, IL-17A, IL-4, IL-5, IL-10, IL-1 and IL-6) at day 0 and month 6.

### Safety data

We will record any adverse medical events in the form of signs, symptoms, abnormal laboratory results or conditions that occur or worsen compared with the baseline. The study sponsor (Nice University Hospital) has purchased insurance for this trial and is responsible for reporting adverse events to the European Medicines Agency.

As rituximab (Truxima®) is an approved treatment by the European Medicines Agency and the US Food and Drug Administration, we do not anticipate significant or serious adverse events. As a result, a data safety monitoring board is not required during the trial.

### Follow-up

Study visits will be performed on day 15, day 30, day 45, month 3, month 6, month 9 and month 12 after the first dose (day 0) of rituximab for routine follow-up.

Clinical assessments (weight, blood pressure, heart rate, presence of oedema and change in concomitant medications) and biosamples will be collected at all follow-up visits, including a shipment to the central laboratory at each follow-up visit.

An individual participant may be discontinued from study intervention in the following

situations:

- Participant decision. The participant is at any time free to discontinue treatment, without prejudice to further treatment.
- An adverse event that, in the opinion of the investigator, warrants discontinuation.

In the event of premature discontinuation of treatment, the patient should be encouraged to comply with protocol evaluations to allow intention-to-treat (ITT) analyses.

### Data collection

The study data will be collected in an e-CRF. This will be implemented by the data manager using the RedCap software. The parameter specification and the implementation of the e-CRF for data collection, including user training, will be provided by the designated data manager.

The investigators and the clinical research assistants at each site will be responsible for collecting and entering data directly into the e-CRF. The data will be securely stored, with specific access rights granted to study teams according to their role.

Data quality control will be performed on the e-CRF, using the patient medical file, by the sponsor during the planned monitoring visits by the Department of Clinical Research and Innovation’s Clinical Research Officer.

Once the final data have been entered, checks for their validity and coherence will be performed by the Data Manager of the Department of Clinical Research and Innovation, and requests for verification will be issued. Throughout the study, any modifications to the database will be recorded, enabling a full audit trail.

At the end of the quality control process, the database will be frozen and signed off by the PI, the data manager and the methodologist. No modification of the data will be possible after this time.

The frozen database, together with the data management report, will then be transferred to the statistician for analysis.

### Data monitoring committee

The data monitoring committee is composed of three research assistants in charge of on-site monitoring, supervised by the iRITUX project manager from the Clinical Research and Innovation Department of the Nice University Hospital.

### Statistical methods

Our analysis strategy will consider two populations.

- ITT population: all patients included in the control or experimental group, whether or not they completed the treatment schedule and had all the biological samples required by the protocol. For the ITT analysis, and in the case of a significant number of lost to follow-up patients (estimated to be 5% for the protocol) that would challenge the robustness of the result, the ‘last observation carried forward’ strategy will be used to impute the missing data for the primary objective.
- Per-protocol (PP) population*:* all patients enrolled in the study who completed the treatment schedule and had all the biological samples required by the protocol.

As indicated in the sample size calculation, three interim efficacy analyses will be performed. The aim of these interim analyses is to demonstrate the positive effect of increasing the rituximab dose according to the patient’s risk level on the remission rate in order to apply this algorithm as soon as possible.

To maintain the alpha risk at 0.025 with a beta risk equal to 0.2 for the final analysis and to limit the possibility of a chance of a positive interim finding, each interim analysis was planned with:

- A one-sided significance level of 0.0002248 was allotted to the first efficacy interim analysis (n=44).
- A one-sided significance level of 0.00209 was allotted to the second efficacy interim analysis (n=66).
- A one-sided significance level of 0.00969 was allotted to the third efficacy interim analysis (n=98).
- A one-sided significance level of 0.02144 was retained for the final analysis (n=130).

The rate of clinical remission observed at 6 months from the first rituximab injection (primary outcome) in the two groups will be compared with access to the significance level, applying a one-sided test for the difference between two binomial proportions (χ^2^ test or Fisher’s exact test in case of a small sample). As the experimental and control groups differ only in the dose of rituximab, with a higher or equivalent dose in the experimental group, a two-sided test is unnecessary in our study.

In case of a statistically significant result at an interim analysis (p value under significance level), the independent committee will decide the termination of the trial (the interim analysis becomes the main analysis). Any retreatment with immunosuppressive therapy (including rituximab) not planned in the protocol before the end of the study at month 12 will be considered a treatment failure (ie, absence of remission). The statistical tests used, parametric or non-parametric, will consider the total number of patients and the distribution of the variables collected.

For secondary objectives, rates of complete and partial clinical remission at month 12 will be compared using a one-tailed test for the difference between two binomial proportions. Changes in biological samples at 3, 6, 9 and 12 months will be evaluated first by analysis of variance if the normal distribution hypothesis is met and second by a paired Student’s t-test or non-parametric Wilcoxon’s S-rank test, as recommended. For longitudinal analyses, secondary outcomes will be analysed using a linear mixed model to account for repeated measures, including fixed effects for time and intercept random effects for patients. The variables of interest and adjustment will be primarily age and sex, given the total number of patients and the distribution of the variables collected.

### Patient and public involvement

Neither patients nor the public were directly involved in the study design or conduct of the study. No plans were established a priori for sharing the results of the study with participants.

## Ethics and dissemination

### Ethical considerations

This study has been approved by the French Ethics Committee (Comité de Protection des Personnes Sud-ouest et Outre-mer), reference number: 1-24-038/24.02335.000451. N° EU-CT: 2024-510718-34-00. This study has also been approved by the French National Agency for Medicines and Health Products Safety and the French National Commission on Informatics and Liberty. The study will be conducted in accordance with the principles of the Declaration of Helsinki, Good Clinical Practice and all applicable regulatory requirements.

Trial registration: NCT06341205 trial number. Registered on April 02, 2024.

### Safety considerations

The benefit/risk balance for this study appears favourable in light of the following.

#### Benefits

##### At the individual level

20%–40% of patients with membranous nephropathy do not respond to a first course of rituximab.^9^ Persistent nephrotic syndrome may be complicated by arterial or venous thrombosis, increased cardiovascular risk, immunodeficiency and chronic renal failure. The persistence of nephrotic syndrome may also affect the quality of life and socioeconomic integration of patients. Patients with nephrotic syndrome have reduced drug bioavailability due to urinary loss of rituximab compared to other non-proteinuric autoimmune diseases treated with rituximab.^11^ We have previously shown that patients with undetectable serum rituximab levels at month 3 were less likely to achieve clinical remission at months 6 and 12.^10^ Additional early doses of rituximab may increase the bioavailability of the drug, increasing the likelihood of early remission by month 6. Repeated doses of rituximab, up to cumulative doses of 3000–6000 mg, according to autoantibody immunomonitoring, have been shown to be effective in inducing clinical remission while being safe.^17 23^ Achieving early remission would help to (1) preserve renal function; (2) preserve patients’ quality of life and socioeconomic integration and (3) limit cardiovascular and thromboembolic risks.

##### At the collective level

Rituximab is a safe and effective treatment for membranous nephropathy.^9 25^ Uncertainty remains about the optimal dose to administer.^26^ The iRITUX algorithm proposes a personalised therapeutic regimen according to the risk of underdosing. If validated, this model will help clinicians maximise the likelihood of remission. This would make it possible to reduce the risk of complications of persistent nephrotic syndrome (eg, thromboembolic complications, cardiovascular morbidity or renal failure), thereby reducing hospitalisations and healthcare costs associated with hospitalisation or sick leave.

### Risks

According to the Summary of Product Characteristics, the most frequently observed adverse drug reactions (ADRs) in patients receiving rituximab were infusion-related reactions which occurred in the majority of patients during the first infusion. The incidence of infusion-related symptoms decreases substantially with subsequent infusions and is less than 1% after eight doses of rituximab.^27^ Infectious events (mainly bacterial and viral) occurred in approximately 30%–55% of patients in the non-Hodgkin’s lymphoma trials and in 30%–50% of patients in the chronic lymphocytic leukaemia trials, but the majority of patients had received rituximab in combination with chemotherapy.^27^ To avoid the risk of infection in our study, patients should have been vaccinated against pneumococcus, SARS-CoV-2 and influenza during the epidemic season. In addition, we have previously shown that patients with membranous nephropathy treated with rituximab during active nephrotic syndrome have reduced bioavailability of rituximab, resulting in less B cell depletion than myasthenic patients treated with rituximab using the same protocol and have a better vaccine response (to SARS-CoV-2 vaccine) than patients treated with rituximab for lymphoid malignancies.^11 28^

The addition of further doses of rituximab should not put patients with membranous nephropathy at greater risk of complications than patients treated for other autoimmune diseases, where the percentage of patients reporting ADRs after retreatment with further courses of rituximab was similar to the percentage of patients reporting ADRs after initial exposure (any grade and grade 3/4 ADRs).^27^ In our protocol, a medical consultation is scheduled for all rituximab injections. During this consultation, a full clinical examination will be carried out to ensure that there are no adverse events.

### Protocol amendments

Any changes to the protocol will be submitted to the regulatory boards. Once approved, the amendments will be immediately communicated by e-mail to the investigators and clinical research assistants at each site. New versions of the amended documents will be placed in the investigator’s site file, and hard copies will be sent to the sites.

### Dissemination

The results will be disseminated in international academic meetings and published in a peer-reviewed journal. The investigators from each centre participating in the trial will be listed as co-authors of the article. Professional writers will not be used to write the article.

## Discussion

Rituximab is recommended by the 2021 KDIGO guidelines and French guidelines as a first-line treatment for primary membranous nephropathy.^7 29^ These recommendations are based on four randomised trials comparing different immunosuppressive regimens in the treatment of membranous nephropathy and a retrospective study comparing adverse events following rituximab or cyclophosphamide therapy. The first trial, GEMRITUX (NCT01508468), compared a low-dose rituximab regimen (375 mg/m^2^ at 1-week interval) with non-immunosuppressive antiproteinuric treatment on the clinical remission rate at month 6. This trial was negative for its primary endpoint but showed a benefit of rituximab after a longer follow-up.^15^ Anti-PLA2R1 antibodies and B cells were not completely depleted 6 months after rituximab treatment, which may indicate that the rituximab regimen used was suboptimal. The second trial, MENTOR (NCT01180036), compared rituximab 1 g at 2-week intervals and oral ciclosporin in achieving clinical remission at 24 months. Rituximab was non-inferior to ciclosporin in inducing clinical remission at 12 months and superior in maintaining clinical remission at 24 months, due to the high rate of clinical relapse with ciclosporin.^30^ The third study, STARMEN (NCT01955187), compared corticosteroid-cyclophosphamide with tacrolimus for 6 months in combination with 1 g of rituximab at month 6. The primary endpoint was clinical remission at 24 months. This study demonstrated the superiority of cyclophosphamide over the combination of tacrolimus and rituximab. Serious adverse events were similar between the two groups.^31^ The last trial, RI-CYCLO (NCT03018535), compared rituximab 1 g at 2-week intervals with corticosteroid-cyclophosphamide. This study did not show a signal of superiority or inferiority of rituximab over a cyclic corticosteroid-cyclophosphamide regimen in the treatment of membranous nephropathy.^32^ To compare the incidence of adverse events in a large cohort, van den Brand *et al* retrospectively compared 100 membranous nephropathy patients who received rituximab with 103 patients treated with corticosteroid-cyclophosphamide. Over a median follow-up of 40 months, the rituximab group had significantly fewer adverse events than the cyclophosphamide group. Although the cumulative incidence of partial remission was lower in the rituximab group, the rates of complete remission and the composite renal endpoint did not differ significantly between groups.^25^ Due to its superior efficacy and safety profile, the KDIGO guidelines and the French guidelines propose the use of rituximab as a first-line treatment in patients with primary membranous nephropathy and active nephrotic syndrome.^7 29^ However, there is still uncertainty about the optimal dosage to be administered. The guidelines do not specify which of the rituximab regimens is to be preferred.^26^ Tailoring the rituximab regimen to patient characteristics is an important challenge.

This study aims to prove the superiority of an algorithm-guided rituximab regimen in patients with primary membranous nephropathy. The results of this study could improve the personalised management of patients with membranous nephropathy and help to better select patients who should benefit from an early reinforced treatment. In fact, the algorithm-driven rituximab regimen could offer an improvement in patient management by providing a personalised therapeutic strategy based on the risk of underdosing estimated as early as day 15. This personalised management could reduce the risk of complications of persistent nephrotic syndrome and improve the patient’s quality of life by increasing the likelihood of clinical remission.

## Supplementary material

10.1136/bmjopen-2024-093920 online supplemental file 1
