# Supplementary material for: Artificial intelligence-based personalised rituximab treatment protocol in membranous nephropathy (iRITUX): protocol for a multicentre randomised control trial
Source: BMJ Open. 2025 Apr 2;15(4):e093920. doi: 10.1136/bmjopen-2024-093920 (PMC11966952; doi:10.1136/bmjopen-2024-093920)
Supplement: online supplemental file 1 [file bmjopen-15-4-s001.doc]

**Artificial Intelligence-based Personalized Rituximab Treatment Protocol in Membranous Nephropathy iRITUX**

# INFORMED CONSENT

I freely agree to participate in this research as described in the information sheet and confirm the following:

- I have had time to read this information, to think about the study and to receive adequate answers to my questions.
- I have been fully informed of the nature of the research objectives, the potential risks and the limitations associated with this research.
- I confirm that I am covered by or benefit from a social security scheme, unless there is an exceptional exemption.
- I have the right to refuse to take part in the research or to withdraw my consent at any time without having to justify my decision, and I will inform the investigator who is following me in the research. This will not affect the quality of my subsequent care.
- I understand that the investigator may interrupt my participation in the research at any time if he or she deems it necessary.
- I understand that I have the right to access, rectify and restrict the processing of my personal data. These rights can be exercised in the first instance with the investigator who is following me as part of this research and who is aware of my identity.
- I authorise access to my directly identifiable personal data to the recipients listed in the information note.
- I am aware that this research is authorised by the CTIS and has been approved by the Agence Nationale de Sécurité du Médicament et des Produits de Santé (ANSM) and has received a favourable opinion from the Comité de Protection des Personnes Sud-Ouest-Outre-Mer I. The research sponsor is insured by RELYENS.
- My consent in no way relieves the investigator and the sponsor of their responsibilities to me. I retain all my rights as guaranteed by law.
- A summary of the research results will be sent to me at the end of the research if I request it from the investigator.
- Once the research has started, I can contact Dr/Pr............................................................................................... for further information at any time.
- Two original copies of this consent form have been made: one has been given to me and the second will be kept by the investigator in the study records for at least 25 years after the end of the research.
- I have been informed of how my personal data and biological samples may be collected, used and shared as described in the information notice.

**I agree that my coded personal data and biological samples may be used for other research related to health or medicine, exclusively for scientific purposes, and I understand that I may withdraw my consent at any time.**

| YES  NO |
| --- |

| **THE PARTICIPANT**  **Last name: .......................................................................................**  **First name: .......................................................................................**  **Date of birth: ...................................................................................** | **Date :** |
| --- | --- |
|  | **Signature :** |
| **THE INVESTIGATOR**  **Last name: ........................................................................................**  **First name: .......................................................................................**  **By signing this form, I certify that I have personally led the discussion, that I have fully informed the participant, that the participant has had sufficient time to make an informed decision, that I have answered the participant's questions and that I am satisfied that the information given has been understood before the participant makes his/her decision.** | **Date :** |
|  | **Signature :** |
| **THE TRUSTED PERSON**  **Last name: .......................................................................................**  **First Name: .......................................................................................**  **By signing this form, I certify that I am acting as an impartial witness and am merely reporting the participant's wishes.** | **Date :** |
|  | **Signature :** |
