# Supplementary material for: Artificial intelligence-based personalised rituximab treatment protocol in membranous nephropathy (iRITUX): protocol for a multicentre randomised control trial
Source: BMJ Open. 2025 Apr 2;15(4):e093920. doi: 10.1136/bmjopen-2024-093920 (PMC11966952; doi:10.1136/bmjopen-2024-093920)
Supplement: Supplementary file 2 [file bmjopen-2024-093920.reviewer_comments.pdf]

PEER REVIEW HISTORY

BMJ Open publishes all reviews undertaken for accepted manuscripts. Reviewers are asked to complete a checklist review form and are provided with free text boxes to elaborate on their assessment. These free text comments are reproduced below.

ARTICLE DETAILS

Title (Provisional)

Artificial Intelligence-based Personalized Rituximab Treatment Protocol in Membranous Nephropathy (iRITUX): Protocol for a Multicentre Randomized Control Trial

Authors

Teisseyre, Maxime; Destere, Alexandre; Cremoni, Marion; Zorzi, Kévin; Brglez, Vesna; Benito, Sylvain; BAILLY, Laurent; Fernandez, Céline; Seitz-Polski, Barbara

---

VERSION 1 - REVIEW

**Reviewer**	**1**
**Name**	**Lam, Thomas Y T**
**Affiliation**	**The Chinese University of Hong Kong, Hong Kong SAR; The Nethersole School of Nursing, Faculty of Medicine, The Chinese University of Hong Kong, Hong Kong SAR.**
**Date**	**04-Nov-2024**
**COI**	**None**

The study presents a personalized Rituximab treatment protocol utilizing a machine learning algorithm, aiming to optimize therapeutic outcomes for membranous nephropathy. I have the following comments.

1. The protocol mentions a "machine learning algorithm" to guide dosing; however, it currently lacks details on the specific type of machine learning model used to construct the algorithm and does not describe the feature engineering process. Providing a comprehensive explanation of the algorithm type, model selection rationale, model comparison and feature engineering steps would strengthen the protocol's methodological transparency. If word count is a problem, author may consider to put the details of machine learning algorithm as appendix.

2. More specific and detailed safety monitoring is needed, particularly the group of patients an underdosing risk above 75% as they will receive a cumulative dose twice than that of the standard of care (control) group.

3. The protocol indicates a difference in total treatment duration across groups, which may confound drug exposure time and total dose. In practical applications, the increased number of doses for high-risk patients may also raise the risk of adverse reactions and impact adherence rates, potentially affecting treatment efficacy. A plan to mitigate these risks—such as through adherence support strategies or closer monitoring—would improve the feasibility and applicability of the protocol.

4. Please justify the assumptions of 50% remission rate of the control group, as well as the 75% remission rate of the intervention group in the sample size estimation.

**Reviewer**	**2**
**Name**	**Schena, Francesco**
**Affiliation**	**Università degli Studi di Bari Aldo Moro**
**Date**	**04-Jan-2025**
**COI**	**None**

The investigators of this multicenter randomised clinical trial have designed a personalised treatment protocol, based on a machine learning algorithm (MLA), to avoid the risk to underdose rituximab in patients with primary membranous nephropathy (MN). The sample size is represented by 130 biopsy-proven MN patients who will be divided into an experimental group (patients with a risk stratified into three levels) and a control group. The time of enrolment is six years and at last one year of observation. Serum samples will be collected during the study to measure some plasma cytokines.

This referee suggests to describe in the paper the type of MLA that will be used (Decision tree, ANN, Random Forest or other).

VERSION 1 - AUTHOR RESPONSE

Reviewer: 1 Dr. Thomas Y T Lam, The Chinese University of Hong Kong, Hong Kong SAR; The Nethersole School of Nursing, Faculty of Medicine, The Chinese University of Hong Kong, Hong Kong SAR.

Comments to the Author:

The study presents a personalized Rituximab treatment protocol utilizing a machine learning algorithm, aiming to optimize therapeutic outcomes for membranous nephropathy. I have the following comments.

Response: We would like to thank Pr. Thomas Y T Lam for his interest in our work and for his important comments, which helped to improve the article. We respond to his comments point by point below.

1. The protocol mentions a "machine learning algorithm" to guide dosing; however, it currently lacks details on the specific type of machine learning model used to construct the algorithm and does not describe the feature engineering process. Providing a comprehensive explanation of the algorithm type, model selection rationale, model comparison and feature engineering steps would strengthen the protocol's methodological transparency. If word count is a problem, author may consider to put the details of machine learning algorithm as appendix.

Response: Thank you for this very important comment. We have added a paragraph 'machine learning algorithm' in the Methods section.

2. More specific and detailed safety monitoring is needed, particularly the group of patients an underdosing risk above 75% as they will receive a cumulative dose twice than that of the standard of care (control) group.

Response: Thank you for that very important comment. Our hypothesis is that the most severe patients receiving 2 g of rituximab will not be exposed to 2 g of effective rituximab due to urinary drug losses. We developed this algorithm to limit the risk of underdosing, and the patients receiving twice the standard dose are the patients with the most severe nephrotic syndrome and therefore the most at risk of underdosing. A previous study showed that higher doses (3 to 6 g) were associated with more frequent remissions without serious adverse events. We have added a paragraph 'Safety consideration' in the Ethics and dissemination section.

3. The protocol indicates a difference in total treatment duration across groups, which may confound drug exposure time and total dose. In practical applications, the increased number of doses for high-risk patients may also raise the risk of adverse reactions and impact adherence rates, potentially affecting treatment efficacy. A plan to mitigate these risks—such as through adherence support strategies or closer monitoring—would improve the feasibility and applicability of the protocol.

Response: Thank you for this very important comment. Patients will be contacted by telephone before each treatment to ensure compliance. A medical consultation is also scheduled for all rituximab injections. During this consultation, a full clinical examination will be carried out to ensure that there are no adverse events. We have added these elements to the methods section. In addition, if the investigator considers that the patient is at risk of non-compliance, that will constitute an exclusion criterion for our study (see page 7).

It should also be noted that in a previous randomised multicentre trial with multiple rituximab infusions (NCT 03804359), we had no problems with compliance.

We have added a paragraph 'Safety consideration' in the Ethics and dissemination section.

4. Please justify the assumptions of 50% remission rate of the control group, as well as the 75% remission rate of the intervention group in the sample size estimation.

Response: Thank you for this very important comment. We based these remission rates on a retrospective study: Rituximab Immunomonitoring Predicts Remission in Membranous Nephropathy - PMC*. In this study, we compared the remission rate between patients who had a rituximab underdosing and patients who did not have an underdosing.*

To set the 50% remission threshold for the standard of care group in our prospective study, we used the overall remission rate observed in our retrospective cohort, which was 53%.

To set the 75% threshold for the personalised treatment group in our prospective study, we based it on the remission rate observed in patients without rituximab underdosing, i.e. around 80%. Indeed, our hypothesis for this group is that algorithm-guided treatment will prevent rituximab underdosing.

We have included a paragraph on this point to the methods section.

Reviewer: 2 Dr. Francesco Schena, Università degli Studi di Bari Aldo Moro

Comments to the Author:

The investigators of this multicenter randomised clinical trial have designed a personalised treatment protocol, based on a machine learning algorithm (MLA), to avoid the risk to underdose rituximab in patients with primary membranous nephropathy (MN). The sample size is represented by 130 biopsy-proven MN patients who will be divided into an experimental group (patients with a risk stratified into three levels) and a control group. The time of enrolment is six years and at last one year of observation. Serum samples will be collected during the study to measure some plasma cytokines.

Response: We would like to thank Pr. Francesco Schena for his interest in our work and for his important comment, which helped to improve the article. We respond to his comment below.

This referee suggests to describe in the paper the type of MLA that will be used (Decision tree, ANN, Random Forest or other).

Response: Thank you for this very important comment. We have added a paragraph 'machine learning algorithm' in the Methods section.

VERSION 2 - REVIEW

**Reviewer**	**1**
**Name**	**Lam, Thomas Y T**
**Affiliation**	**The Chinese University of Hong Kong, Hong Kong SAR; The Nethersole School of Nursing, Faculty of Medicine, The Chinese University of Hong Kong, Hong Kong SAR.**
**Date**	**18-Feb-2025**
**COI**	

Thank you for the revised manuscript. All comments have adequately addressed. I have no furether comments.
